# Effectiveness and Safety of Nirmatrelvir/Ritonavir (Paxlovid) Versus Remdesivir in COVID‐19: A Systematic Review and Meta‐Analysis of Retrospective Studies

**DOI:** 10.1002/hsr2.73316

**Published:** 2026-09-30

**Authors:** Arash Akbarzadeh, Masoud Razeghian, Foruzan Hajiabadi, Parvaneh Dehghan, Sadegh Ahmadi‐Mazhin, Maliheh Eshaghzadeh, Mohsen Poursadeqiyan, Rouhollah Shabestan

**Affiliations:** ^1^ Department of Biostatistics and Epidemiology, School of Public Health Tehran University of Medical Sciences Tehran Iran; ^2^ School of Medicine Torbat Heydariyeh University of Medical Sciences Torbat Heydariyeh Iran; ^3^ School of Nursing and Midwifery University of Medical Sciences Shiraz Iran; ^4^ Department of Oncology, School of Medicine Torbat Heydariyeh University of Medical Sciences Torbat Heydariyeh Iran; ^5^ Department of Public Health, School of Health Ahvaz Jundishapur University of Medical Sciences Ahvaz Iran; ^6^ Department of Medical Surgical Nursing, School of Nursing and Midwifery Torbat Heydariyeh University of Medical Sciences Torbat Heydariyeh Iran; ^7^ Social Determinants of Health Research Center, School of Health Ardabil University of Medical Sciences Ardabil Iran

**Keywords:** COVID‐19, effectiveness, nirmatrelvir/ritonavir, paxlovid, remdesivir, safety, SARS‐CoV‐2

## Abstract

**Background and Aim:**

Nirmatrelvir/ritonavir (Paxlovid) and remdesivir are antiviral agents that have been widely used in the management of patients with Coronavirus disease 2019 (COVID‐19). This systematic review and meta‐analysis were conducted to compare the clinical effectiveness and safety outcomes of Paxlovid and remdesivir among patients with COVID‐19.

**Methods:**

A systematic search was performed across the Cochrane Library, Web of Science, PubMed, and medRxiv from inception to July 2024. Data from the identified studies were analyzed using Comprehensive Meta‐Analysis (CMA) software.

**Results:**

Thirteen studies involving 4583 patients were included in the final analysis. Compared with remdesivir, Paxlovid was associated with a significant reduction in mortality (odds ratio [OR] = 0.36, 95% confidence interval [CI]: 0.18–0.73), time to SARS‐CoV‐2 negative conversion (standardized mean differences [SMD] = −1.44, 95% CI: −1.54 to −1.33), hospitalization (OR = 0.34, 95% CI: 0.16–0.74), intensive care unit admission (OR = 0.10, 95% CI: 0.01–0.53), and the need for oxygen therapy (OR = 0.06, 95% CI: 0.02–0.16). No statistically significant difference was observed between the two treatments regarding polymerase chain reaction negativity rate (OR = 1.45, 95% CI: 0.73–2.86). However, adverse events were higher among patients receiving Paxlovid (OR = 6.60, 95% CI: 3.24–13.42). The certainty of evidence for the evaluated outcomes ranged from low to moderate.

**Conclusion:**

In patients with COVID‐19, Paxlovid may offer potential benefits over remdesivir for certain clinical outcomes, though it may also be accompanied by a higher rate of adverse events. These findings are based on retrospective studies, which carry risks of selection bias, confounding, and heterogeneity.

## 1 Introduction

Vaccination has played an important role in protecting individuals against severe forms of Coronavirus disease 2019 (COVID‐19) caused by the severe acute respiratory syndrome coronavirus 2 (SARS‐CoV‐2) virus [1]. However, some populations remain at increased risk of severe outcomes, including morbidity and mortality, following SARS‐CoV‐2 infection. Therefore, additional effective treatment strategies are still needed, particularly for vulnerable individuals [2, 3, 4]. Evidence suggests that antiviral therapies may provide additional benefits when used alongside vaccination. This combined approach could contribute to better control of COVID‐19 and its complications [5]. These findings highlight the importance of antiviral agents as part of a broader approach to COVID‐19 management, particularly when combined with vaccination strategies, to reduce disease burden, hospitalization, and mortality [5]. Several antiviral agents have been investigated and used for the treatment of COVID‐19, including nirmatrelvir/ritonavir (Paxlovid) [6], remdesivir [7], molnupiravir [8], and Azvudine [9]. The U.S. Food and Drug Administration (FDA) has approved Paxlovid for treating mild‐to‐moderate COVID‐19 in adults at high risk for severe outcomes, including hospitalization or death [10]. Additionally, the FDA approved Veklury) remdesivir) for treatment in both adult and pediatric patients aged 12 years and older who weigh at least 40 kilograms and require hospitalization due to COVID‐19 [11]. Previous meta‐analyses of real‐world studies and randomized clinical trials have demonstrated the effectiveness of these antiviral agents compared with standard care in improving clinical outcomes among patients with COVID‐19 [7, 12].

However, real‐world studies directly comparing the effectiveness of these therapies against SARS‐CoV‐2 have reported inconsistent findings [13, 14, 15]. Some research indicates a superior effectiveness of Paxlovid over remdesivir [13, 14], while other investigations report contrary findings [15, 16, 17]. Given the lack of evidence from a systematic review and meta‐analysis directly comparing these two agents, this study aimed to evaluate the effectiveness and safety of Paxlovid versus remdesivir in patients with COVID‐19.

## 2 Methods

The protocol for this systematic review and meta‐analysis was not registered in any protocol registration database. The Preferred Reporting Items for Systematic Reviews and Meta‐Analyses (PRISMA) guidelines were followed in the preparation and reporting of this systematic review [18] (Supporting Information S1: Table S1).

### 2.1 Literature Search

A comprehensive literature search was conducted in PubMed, the Cochrane Library, Web of Science, medRxiv, and Google Scholar to identify relevant studies comparing nirmatrelvir/ritonavir (Paxlovid) and remdesivir in patients with COVID‐19 up to July 2024. In addition to the database search, the reference lists of all included studies were manually reviewed to identify any additional relevant articles that might not have been retrieved through the initial search process. The search was conducted without restricting studies based on language. The search terms included a combination of relevant keywords and phrases, including, but not limited to COVID‐19, SARS‐CoV‐2, nirmatrelvir/ritonavir, Paxlovid, and remdesivir. To facilitate transparency and reproducibility, the complete search strategies applied for each database are presented in the supporting material of this study.

### 2.2 Study Selection

The study selection process was conducted based on predefined inclusion and exclusion criteria according to the objectives of this review. Studies were eligible for inclusion if they included patients with COVID‐19 confirmed by polymerase chain reaction (PCR) testing and evaluated treatment with either Paxlovid or remdesivir. In addition, eligible studies were required to assess relevant effectiveness and safety outcomes, including mortality, hospitalization, and treatment‐related adverse events. Studies investigating combination therapies, other forms of interventions, reporting case reports, evaluating outcomes unrelated to the objectives of this review, or published as letters to the editor were excluded.

### 2.3 Risk of Bias and Certainty of Evidence

The risk of bias of the included non‐randomized studies was independently evaluated using the Risk Of Bias In Non‐randomized Studies of Interventions (ROBINS‐I) tool [19]. The assessment covered seven domains: confounding, selection of participants, classification of interventions, deviations from the intended interventions, missing data, measurement of outcomes, and selective reporting of results. Each domain was evaluated using the signaling questions provided in the ROBINS‐I framework and was subsequently rated as having a low, moderate, serious, or critical risk of bias, or as having insufficient information to make a judgment. When the two assessors disagreed, the issue was discussed until consensus was reached; if consensus could not be achieved, a third author was consulted. The certainty of the evidence for the assessed outcomes was also examined using the Grading of Recommendations Assessment, Development, and Evaluation (GRADE) approach. Certainty was judged by considering the risk of bias, inconsistency, indirectness, and imprecision of the available evidence.

### 2.4 Data Extraction

Relevant data were independently extracted by two authors using a standardized data extraction form. The extracted information included the following domains: (1) study characteristics, including the first author's name, year of publication, study location, and study design; (2) patient characteristics, including the total number of participants, sex distribution, mean age, presence of comorbidities, and COVID‐19 vaccination status; (3) treatment characteristics, including sample size, mean age of participants, administered dosage, and treatment duration; (4) effectiveness outcomes, including mortality, hospitalization, PCR negativity rate, and time to SARS‐CoV‐2 negative conversion; and (5) safety outcomes, including the frequency of reported adverse events.

### 2.5 Data Analysis

Statistical analyses were carried out with Comprehensive Meta‐Analysis (CMA) version 3.0. For dichotomous outcomes, odds ratios (ORs) with 95% confidence intervals (CIs) were calculated, whereas continuous outcomes were analyzed using standardized mean differences (SMDs) with 95% CIs. Statistical heterogeneity was assessed using the *I* ^2^ statistic and Cochran's Q test. Substantial heterogeneity was considered present when *I* ^2^ exceeded 50%, or the Q test showed a *p* value < 0.10. A random‐effects model was applied when significant heterogeneity was detected, while a fixed‐effect model was used for analyses without substantial heterogeneity. A leave‐one‐out sensitivity analysis was conducted to assess the robustness of the pooled results by removing one study at a time and evaluating its impact on the overall estimates. Effect estimates (ORs and SMDs) are presented with 95% CIs to describe both the magnitude and precision of the findings. *p* values were reported alongside effect estimates and confidence intervals.

## 3 Results

### 3.1 Search Findings

The flow diagram illustrating the process of identification, screening, and selection of studies based on title, abstract, and full text is presented in Figure 1. Initially, a comprehensive search yielded a total of 235 studies that were identified as potentially relevant to the research topic. After removing duplicate records, the remaining studies underwent title and abstract screening, resulting in 105 studies being assessed for eligibility. Of these, 86 studies were excluded based on the predefined inclusion criteria. Following this initial screening, the full text of the remaining 19 studies was carefully reviewed in detail. The full texts of the remaining 19 studies were then evaluated in detail, and 13 studies that fulfilled the eligibility criteria were included in the final analysis [13, 14, 15, 16, 20, 21, 22, 23, 24, 25, 26, 27, 28]. All included studies were retrospective designs. The mean age of participants across the included studies ranged from 8 to 72 years, representing a diverse patient population. The main characteristics of the included studies are summarized in Table 1.

**Figure 1:**
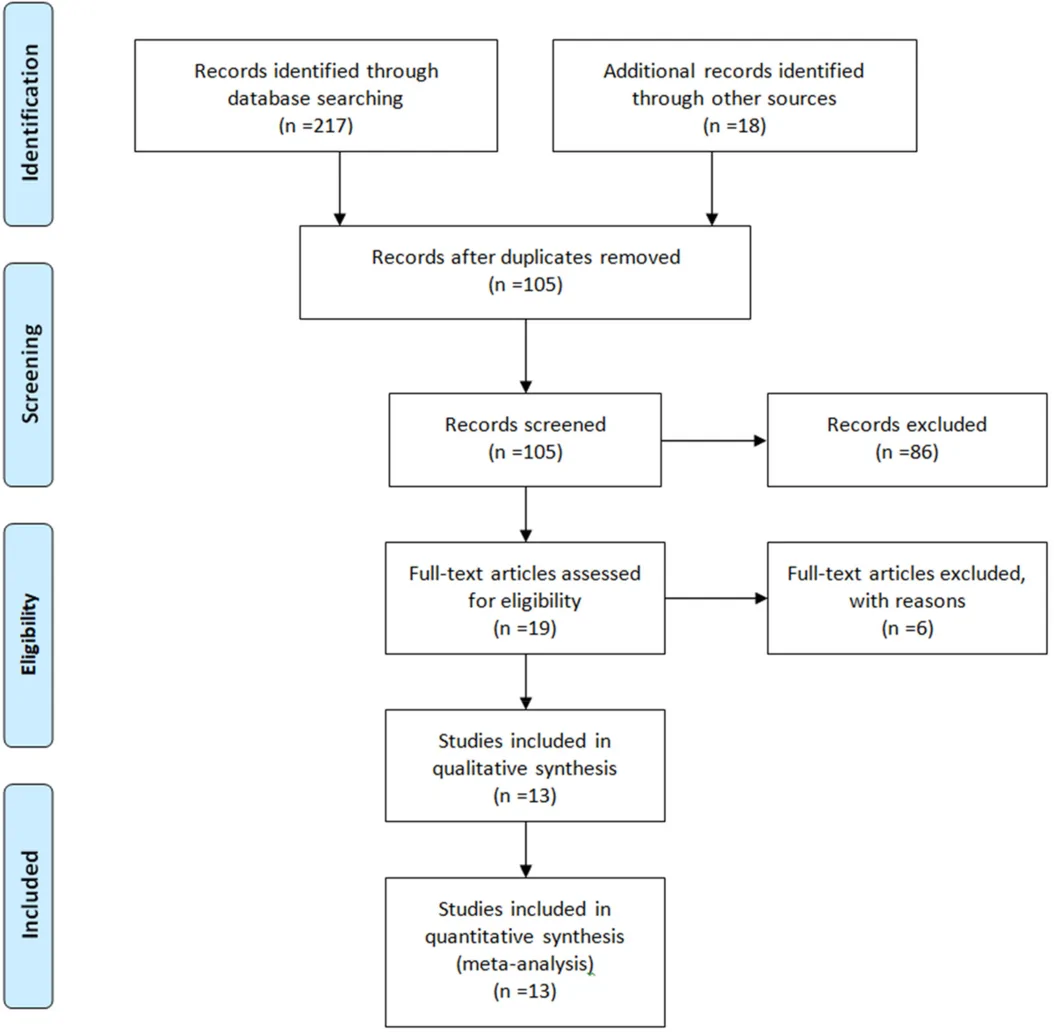
Flow diagram of PRISMA.

**Table 1:** Characteristics of studies included in the systematic review and meta‐analysis.

Study	Year	Place	Paxlovid					Remdesivir					Follow‐up (days)
			Mean age	*N*	Male	Comorbidity(%)^a^	Vaccination rate (%)^b^	Mean age	*N*	Male	Comorbidity(%)^a^	Vaccination rate (%)^b^	
Bai et al. [13]	2024	Italy	70	150	80	22.7	80.7	78	134	56	43.3	91	NA
Basoulis et al. [16]	2023	Greece	65.2	165	100	46.7	89.8	60.2	356	198	60.6	88	30
Borgo et al. [15]	2023	Italy	64	398	NR	45	93.8	66	230	NR	56.5	86.1	30
Celorio et al.[24]	2024	Spain	62	94	35	NA	85.1	62	124	54.8	NA	83.9	30
Exquis et al. [28]	2024	Switzerland	NA	180	NA	NA	NA	NA	78	NA	NA	NA	NA
Lahouati et al. [20]	2023	France	66	49	28	32	61	63	10	80	60	100	30
Lasagna et al. [21]	2022	Italy	NR	34	10	50	100	NR	5	1	40	80	NA
Lopez et al. [14]	2024	Mexico	64	332	110	42.8	96.8	55	451	186	51.2	94.5	28
Manciulli et al. [22]	2023	Italy	66.9	120	51	47.5	97.5	67.4	142	41.6	55.6	88	28
Piñana et al. [23]	2023	Spain	65	223	131	35	94	64	243	150	43	91	NA
Rinaldi et al. [25]	2023	Italy	67	360	180	65.6	88.4	64	207	119	68.8	94	NA
Tiseo et al. [26]	2023	Italy	65	252	127	69	86.9	72	196	113	86.7	77	30
Vora et al. [27]	2022	USA	14.5	12	NA	NA	NA	8	38	NA	NA	NA	NA

Abbreviations: *N*, number; NA, not acquired.

a Having at least one comorbidity.

b Receipt of ≥1 dose SARS‐CoV‐2 vaccine.

### 3.2 Risk of Bias

The result of risk of bias assessment using the ROBINS‐I tool is presented in Supporting Information S1: Table S2. The quality of included studies was acceptable.

### 3.3 GRADE

The results of the GRADE assessment for the main outcomes are presented in the Summary of Findings table (Supporting Information S1: Table S3). The evidence was rated as moderate for mortality rate, hospitalization rate, ICU admission, and oxygen therapy, and low for SARS‐CoV‐2 negative conversion time, PCR rate, and adverse events. Downgrades were primarily due to serious risk of bias in the retrospective cohort studies, inconsistency, and imprecision. No serious concerns were noted for indirectness or other domains.

### 3.4 Effectiveness Outcomes

#### 3.4.1 Mortality Rate

Eight studies [13, 14, 15, 16, 22, 23, 25, 26] involving 3909 patients assessed the mortality rate in patients with COVID‐19. The integrated analysis showed a significant difference between the Paxlovid and remdesivir groups in terms of mortality rate (OR = 0.36, 95% CI: 0.18–0.73; *p* < 0.001, *I* ^2 ^= 6%, GRADE certainty: moderate) (Figure 2).

**Figure 2:**
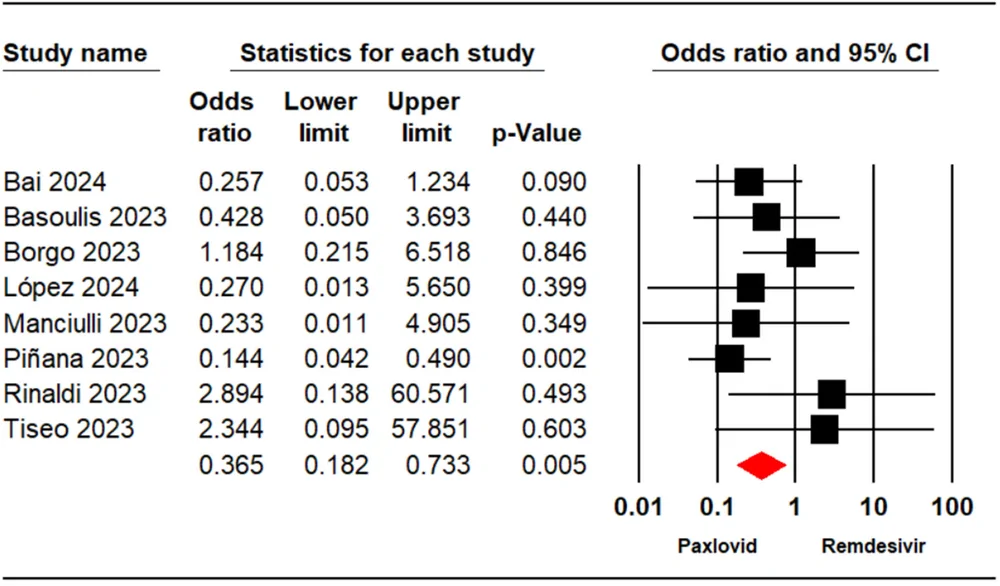
Forest plot of mortality rate between nirmatrelvir/ritonavir (Paxlovid) and remdesivir.

#### 3.4.2 SARS‐CoV‐2 Negative Conversion Time

Five studies [13, 15, 23, 25, 26] involving 1947 patients reported time to SARS‐CoV‐2 negative conversion in patients with COVID‐19. The integrated analysis showed a significant difference between the Paxlovid and remdesivir groups in terms of SARS‐CoV‐2 negative conversion time (SMD = −1.44, 95% CI: − 1.54 to −1.33; *p* < 0.001, *I* ^2 ^= 98%, GRADE certainty: low) (Figure 3).

**Figure 3:**
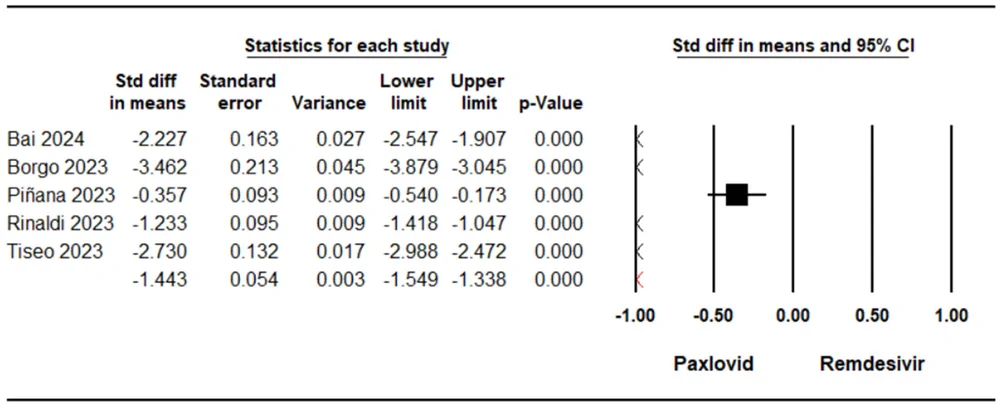
Forest plot of SARS‐CoV‐2 negative conversion time between nirmatrelvir/ritonavir (Paxlovid) and remdesivir.

#### 3.4.3 Hospitalization Rate

Nine studies [14, 16, 20, 22, 23, 24, 25, 26, 27] assessed hospitalization rate in 3374 patients. The pooled estimate indicated a significant difference was observed between the Paxlovid and remdesivir groups in terms of hospitalization rate (OR = 0.34, 95% CI: 0.16–0.74; *p* < 0.001, *I* ^2 ^= 68%, GRADE certainty: moderate) (Figure 4).

**Figure 4:**
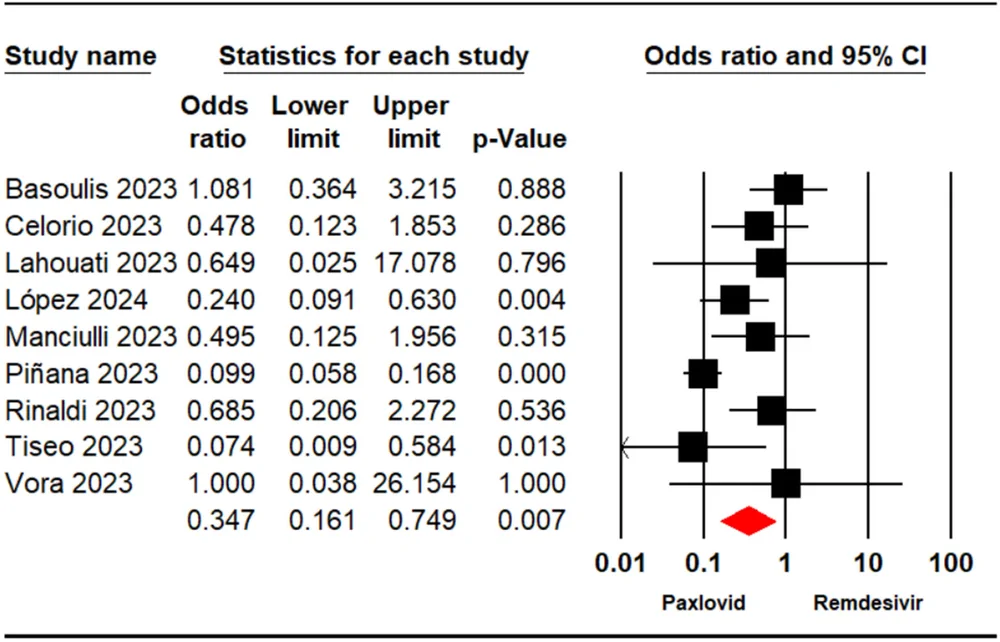
Forest plot of hospitalization rate between nirmatrelvir/ritonavir (Paxlovid) and remdesivir.

#### 3.4.4 PCR Rate

Four studies [13, 23, 25, 26] involving 1675 patients reported PCR rate among COVID‐19 patients treated with Paxlovid and remdesivir. The integrated analysis revealed no significant difference between the Paxlovid and remdesivir groups in terms of PCR rate (OR = 1.45, 95% CI: 0.73–2.86; *p* = 0.27, *I* ^2 ^= 85%, GRADE certainty: low) (Figure 5).

**Figure 5:**
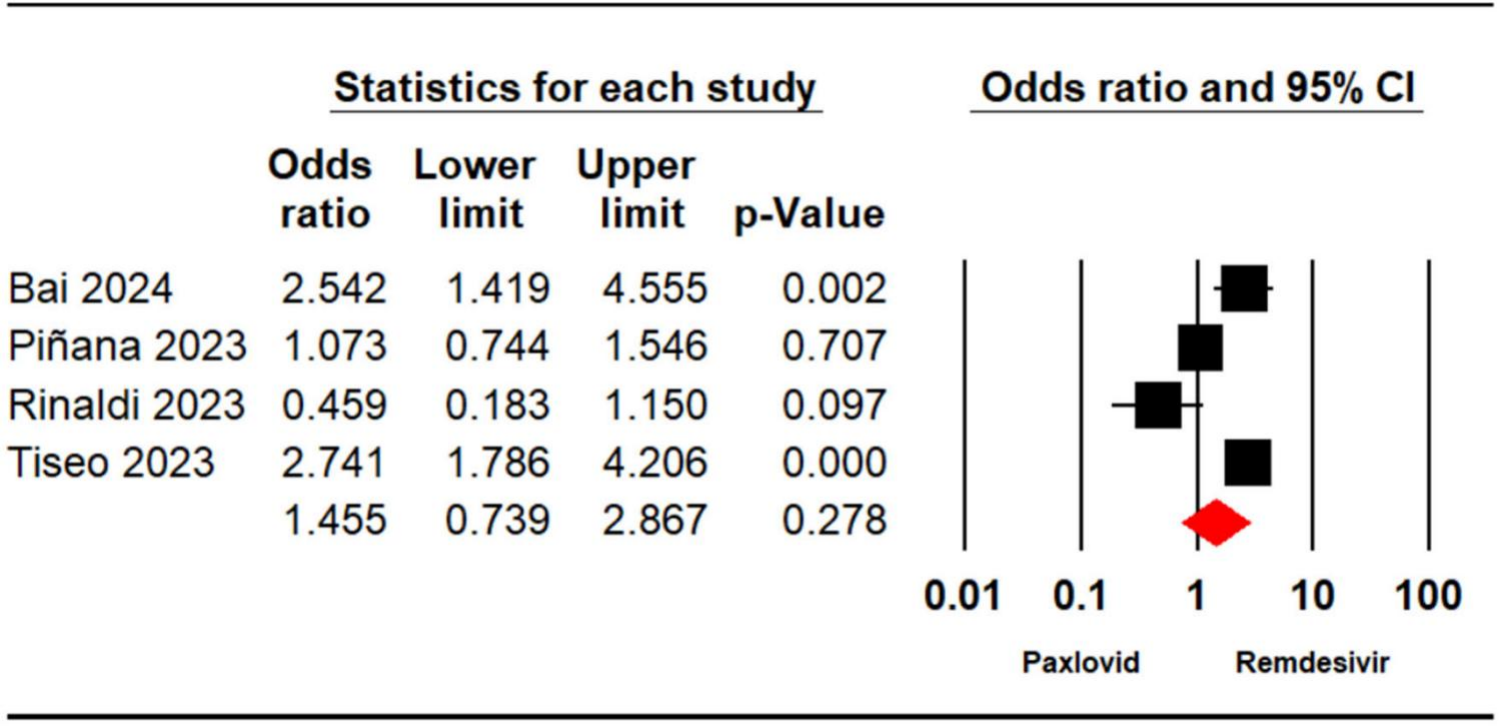
Forest plot of PCR rate between nirmatrelvir/ritonavir (Paxlovid) and remdesivir.

#### 3.4.5 ICU Admission

Two studies [20, 23] involving 525 patients reported ICU admission among COVID‐19 patients treated with Paxlovid and remdesivir. The integrated analysis revealed a significant difference between the Paxlovid and remdesivir groups in terms of ICU admission (OR = 0.10, 95% CI: 0.01–0.53; *p* < 0.001, *I* ^2 ^= 0%, GRADE certainty: moderate) (Figure 6).

**Figure 6:**
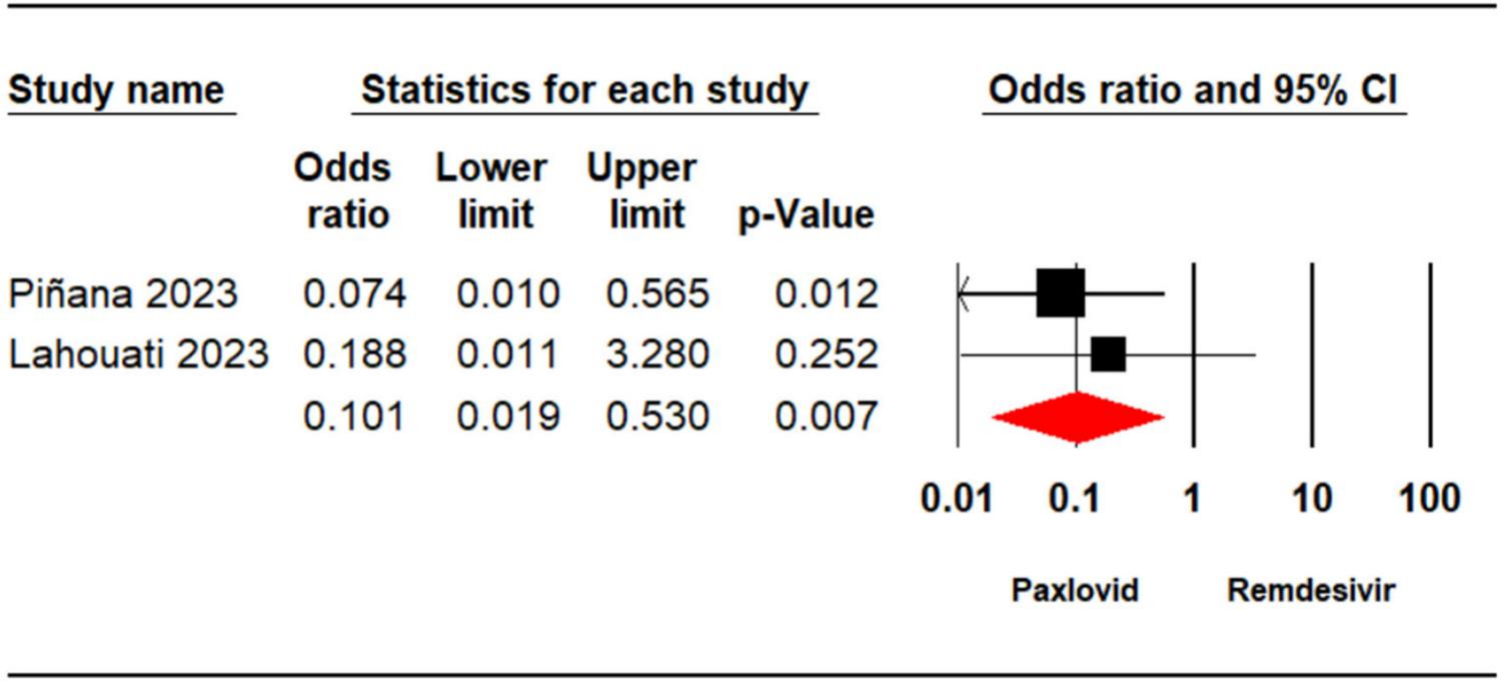
Forest plot of ICU admission between nirmatrelvir/ritonavir (Paxlovid) and remdesivir.

#### 3.4.6 Oxygen Therapy

Two studies [20, 23] assessed oxygen therapy in 525 patients. The pooled estimate indicated a significant difference was observed between the Paxlovid and remdesivir groups in terms of oxygen therapy (OR = 0.06, 95% CI: 0.02–0.16; *p* < 0.001, *I* ^2 ^= 48%, GRADE certainty: Moderate) (Figure 7).

**Figure 7:**
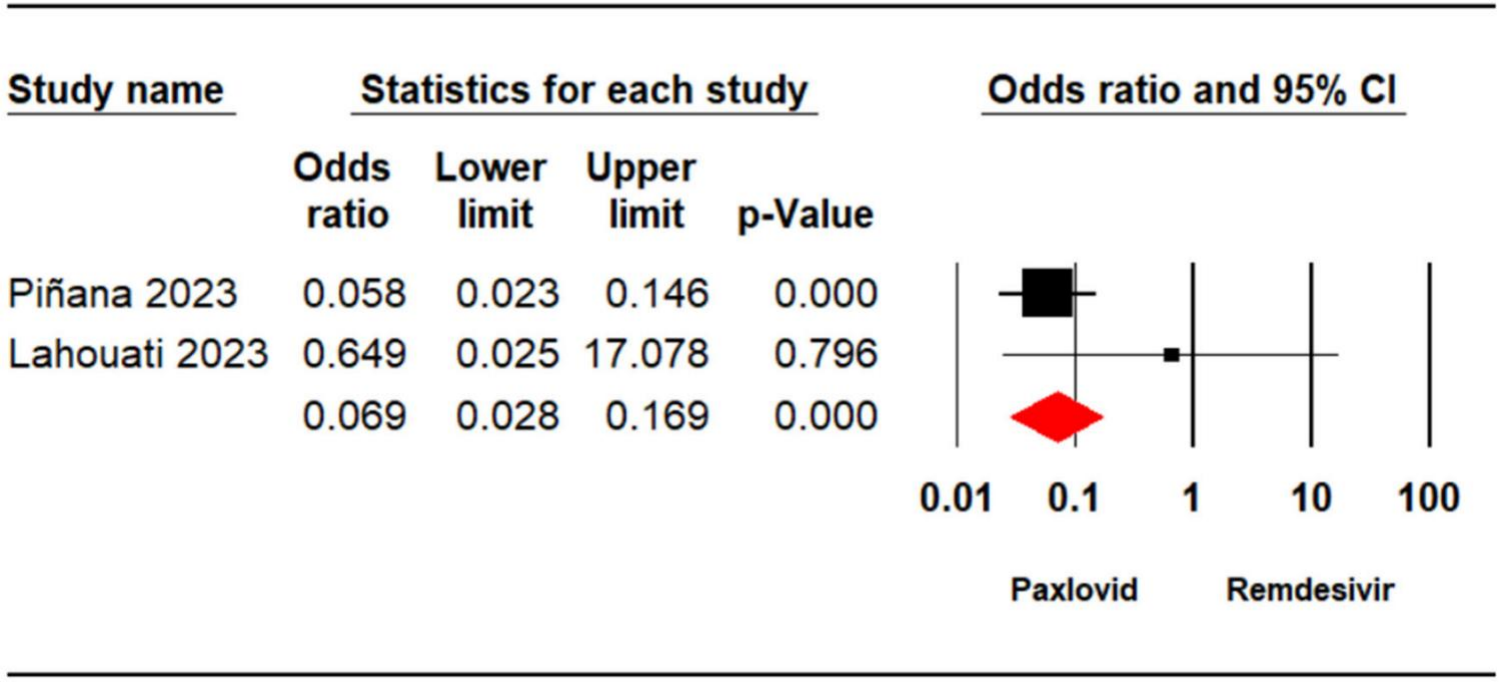
Forest plot of oxygen therapy between nirmatrelvir/ritonavir (Paxlovid) and remdesivir.

### 3.5 Safety Outcomes

#### 3.5.1 Adverse Events

Meta‐analysis of nine studies [13, 15, 21, 22, 23, 24, 25, 26, 28] with 3071 patients demonstrated that there was a significant difference observed between the Paxlovid and remdesivir groups in terms of adverse events (OR = 6.60, 95% CI: 3.24–13.42; *p* < 0.001, *I* ^2 ^= 70%, GRADE certainty: low) (Figure 8).

**Figure 8:**
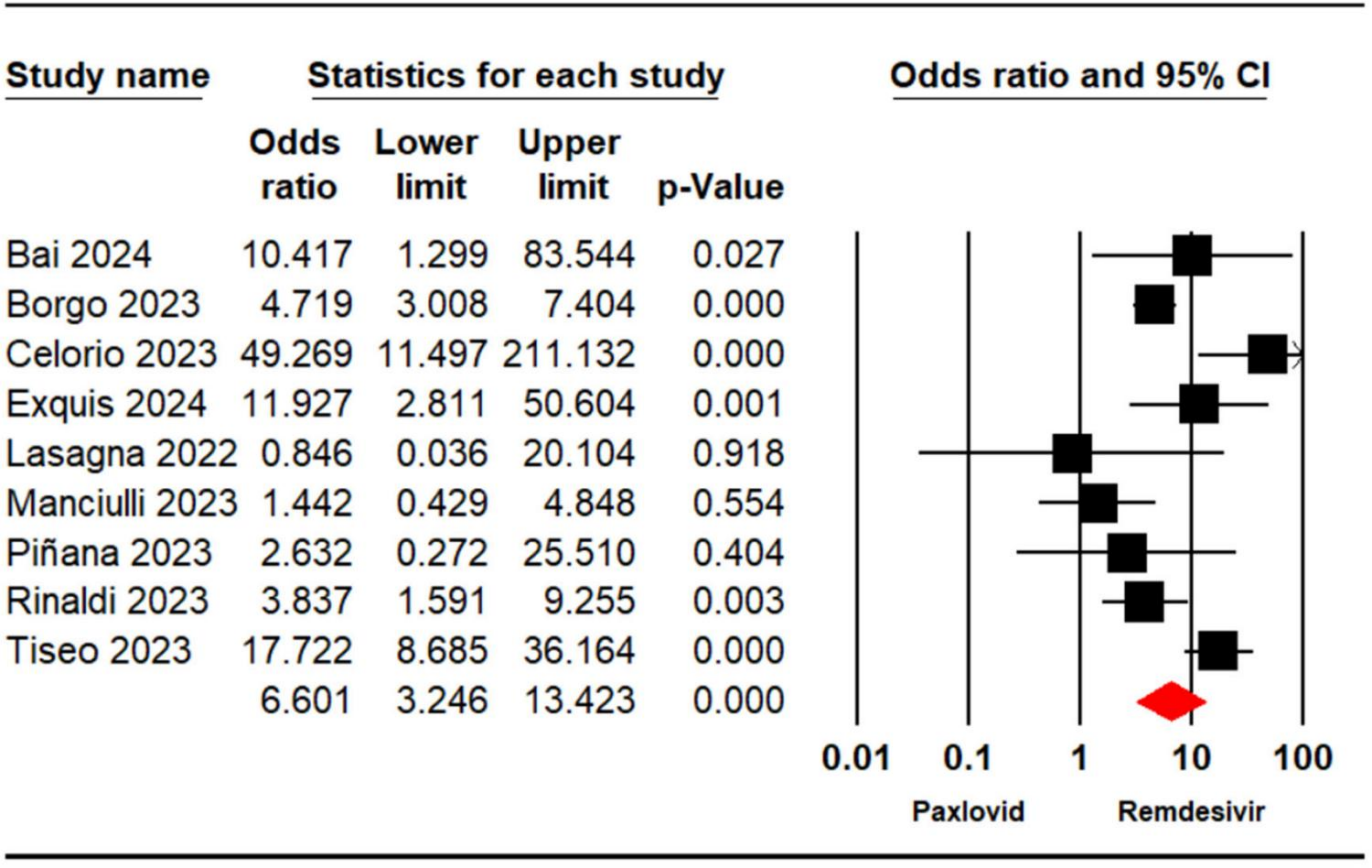
Forest plot of adverse events between nirmatrelvir/ritonavir (Paxlovid) and remdesivir.

### 3.6 Subgroup and Sensitivity Analyses

Sensitivity analysis using the leave‐one‐out method revealed no substantial changes in the pooled estimates compared to the primary analysis across all outcomes, including mortality rate, SARS‐CoV‐2 negative conversion time, hospitalization rate, PCR rate, ICU admission, oxygen therapy, and adverse events (Supporting Information S1: Figures S1–S6).

## 4 Discussion

Considering the limited protection offered by some COVID‐19 vaccines in certain individuals, it is crucial to explore and enhance antiviral treatment options for this vulnerable population to bolster overall immunity and protect public health [29]. Evidence has shown that several antiviral agents and monoclonal antibodies have been beneficial in improving clinical outcomes in patients with COVID‐19 [8, 12, 30, 31, 32].

Pooled estimate of studies showed that Paxlovid was more effective than remdesivir in reducing mortality rates among COVID‐19 patients. This advantage may stem from Paxlovid's superior effectiveness in preventing the progression of the disease to severe stages [16]. While remdesivir has shown some effectiveness in decreasing mortality compared to placebo [33], its overall effect on mortality rates in COVID‐19 patients remains inconsistent across studies [33, 34]. Some meta‐analyses suggest that remdesivir may significantly reduce the risk of death among patients with COVID‐19 [34, 35], while others have reported no clinical benefits associated with its use [33, 36]. However, recent meta‐analyses showed no difference between remdesivir with molnupiravir or sotrovimab in reducing COVID‐19‐related mortality [37, 38]. Conversely, the evidence supporting Paxlovid's effectiveness in reducing mortality, particularly among high‐risk groups, appears to be more robust [39].

When comparing the real‐world studies that directly evaluate the effectiveness of Paxlovid and remdesivir in reducing COVID‐19‐related deaths, Paxlovid was associated with a lower mortality rate among patients with COVID‐19 compared with remdesivir [15, 23].

For instance, a study by Piñana revealed that patients with COVID‐19 treated with Paxlovid had a significantly lower mortality rate than those who received remdesivir [23]. Moreover, a study conducted by Manciulli et al. [22] reported no deaths among patients with COVID‐19 treated with Paxlovid, compared with a 1.4% mortality rate among those receiving remdesivir. The meta‐analysis showed that Paxlovid was associated with a significantly lower hospitalization rate among patients with COVID‐19 compared with remdesivir. This difference may be explained by several factors, including the timing of treatment initiation [40]. Paxlovid is recommended for outpatient treatment within 5 days of symptom onset, when viral replication is more active, whereas remdesivir is generally administered in hospitalized patients, potentially after disease progression has occurred [40]. Evidence from previous studies showed that patients who received Paxlovid within 5 days of diagnosis had a 51% lower risk of hospitalization within 30 days compared with those who did not receive treatment [41]. Several studies have highlighted that early administration of Paxlovid may reduce the risk of hospitalization and viral rebound, emphasizing the importance of timely treatment compared with remdesivir, which is usually initiated after disease progression [40, 41, 42]. Most studies directly comparing these treatments have reported lower hospitalization rates with Paxlovid than remdesivir [25, 26]. However, meta‐analyses showed the comparable effectiveness of remdesivir versus molnupiravir or sotrovimab in reducing COVID‐19‐related hospitalization [37, 38].

Moreover, the meta‐analysis showed that the time to SARS‐CoV‐2 negative conversion was significantly shorter in patients treated with Paxlovid compared with those receiving remdesivir. However, no significant difference was observed between the two treatments in the rate of negative PCR results. The faster viral clearance observed with Paxlovid may be related to its early administration, which occurs during the phase of active viral replication [40]. Previous studies have shown that Paxlovid significantly reduces the time required to achieve SARS‐CoV‐2 negativity compared with no antiviral treatment [43, 44]. Although remdesivir has also been associated with a shorter duration of viral positivity [45], the evidence supporting Paxlovid's effect on viral clearance is more extensive, whereas data regarding remdesivir remain limited [46]. In studies directly comparing these two agents, Paxlovid has generally been associated with faster SARS‐CoV‐2 negative conversion and higher rates of negative PCR results than remdesivir [15, 26].

Additionally, the meta‐analysis showed that Paxlovid was associated with significantly lower ICU admission rates and reduced oxygen therapy requirements compared with remdesivir. Previous studies have also supported the effectiveness of both treatments in reducing ICU admissions and the need for oxygen therapy among patients with COVID‐19 [47, 48, 49].

A meta‐analysis by Tian et al. [47] reported that Paxlovid was associated with lower ICU admission rates and reduced oxygen therapy requirements compared with no Paxlovid treatment.

Similarly, Boglione et al. found that remdesivir was associated with a lower risk of ICU admission compared with the control group (9.8% vs. 17.8%) [48]. Furthermore, Alsayed et al. reported that early administration of remdesivir was associated with fewer ICU admissions and a lower need for mechanical ventilation among hospitalized patients with COVID‐19 [50].

The meta‐analysis showed a higher incidence of adverse events among patients treated with Paxlovid compared with those receiving remdesivir. Previous meta‐analyses have reported higher rates of adverse events in COVID‐19 patients treated with Paxlovid compared with those not receiving Paxlovid [47, 51]. In contrast, other meta‐analyses found no significant difference in adverse events between remdesivir and no remdesivir treatment [34, 52]. However, potential severe adverse events associated with these antiviral therapies remain an important concern [34, 36]. Some studies have reported possible drug‐drug interactions in patients receiving Paxlovid, particularly among those taking medications for other underlying conditions [53]. In contrast, remdesivir treatment has been associated with increased liver enzyme levels in patients with COVID‐19 [54]. Therefore, careful monitoring for drug interactions and liver toxicity is recommended in patients treated with either Paxlovid or remdesivir.

This study has several limitations that should be considered. First, the inclusion of retrospective studies in our systematic review and meta‐analysis may have introduced biases that could affect the reliability of the findings. These study designs are associated with an increased risk of selection bias, confounding by indication, and reporting bias. Second, differences among study populations, including COVID‐19 vaccination rates, comorbidity prevalence, and disease severity, may have contributed to heterogeneity. However, subgroup analyses based on these factors could not be performed due to insufficient data from the included studies. Third, the presence of multiple treatment groups in several studies may have complicated the data analysis. The variability in patient characteristics and clinical settings may also have contributed to substantial heterogeneity. Therefore, the pooled estimates should be interpreted with caution.

## 5 Conclusion

This study suggests that Paxlovid may provide greater clinical benefits than remdesivir in patients with COVID‐19, although it may be associated with a higher risk of adverse events. However, these findings should be interpreted with caution due to the low‐to‐moderate certainty of evidence and possible differences in effectiveness across emerging SARS‐CoV‐2 variants. Further high‐quality studies are needed to confirm these results and evaluate the cost‐effectiveness of both treatments in different healthcare settings.

## Author Contributions

**Arash Akbarzadeh:** methodology, conceptualization, supervision, project administration, writing – review and editing, formal analysis, resources. **Masoud Razeghian:** validation, writing – original draft, writing – review and editing. **Foruzan Hajiabadi:** validation, formal analysis, writing – review and editing, software. **Parvaneh Dehghan:** validation, software, data curation. **Sadegh Ahmadi‐Mazhin:** validation, writing – original draft, writing – review and editing, software, resources, visualization. **Maliheh Eshaghzadeh:** conceptualization, methodology, data curation, visualization, software, resources. **Mohsen Poursadeqiyan:** writing – original draft, writing – review and editing, methodology, conceptualization, resources. **Rouhollah Shabestan:** conceptualization, methodology, formal analysis, writing – original draft, writing – review and editing, project administration, supervision, investigation, validation.

## Funding

The authors have nothing to report.

## Conflicts of Interest

The authors declare no conflicts of interest.

## Transparency Statement

Rouhollah Shabestan affirms that this manuscript is an honest, accurate, and transparent account of the study being reported; that no important aspects have been omitted; and that any discrepancies from the study as planned have been explained.

## Supporting information


Supporting File

## Data Availability

The data that support the findings of this study are available from the corresponding author upon reasonable request.
